# Relational Agency of University Teachers of Chinese as a Second Language: A Personal Network Perspective

**DOI:** 10.3389/fpsyg.2021.790592

**Published:** 2022-01-06

**Authors:** Weijia Yang, Citing Li, Xuesong Gao

**Affiliations:** ^1^School of English Studies, Shanghai International Studies University, Shanghai, China; ^2^Faculty of Education, University of New South Wales, Sydney, NSW, Australia

**Keywords:** Chinese as a second language (CSL), relational agency, personal network, relational resources, professional development

## Abstract

Relational agency is pivotal for understanding how language teachers seek and utilize relational resources in different contexts and grow to be agents of change amid various educational challenges. This study explored how three university teachers of Chinese as a second language (CSL) enacted their relational agency to enhance their research capacity and sustain their professional development. Data on their personal network development was collected through concentric circle interviews, life-history interviews and written reflections over three months. Thematic analysis was adopted for iterative coding and interpretation of the data. The findings revealed that teachers’ personal networks provided them with value guidance, emotional support and academic support, which exerted differential levels of impact on them to make agentic choices and actions. The study suggests that personal network analysis may serve as a suitable theoretical lens to achieve a multi-layered understanding of relational agency. The study also calls for more efforts to create learning opportunities and spaces in the relational context for teachers to build their career as agentic academics in language teacher education and development programs.

## Introduction

The notion of teacher agency as teachers’ “socioculturally mediated capacity to act” (Ahearn, 2001, p. 112) has provided a critical lens for researchers to explore and understand how university teachers invested efforts in “(building) their career as academics” (Tao et al., 2020, p.13). Research has examined the critical role that teacher agency has in initiating and sustaining teachers’ professional development. As teacher agency often occurs through interactive positionings and social interactions depending upon others, it is relational in nature and sometimes shared (e.g., Edwards, 2005; Kayi-Aydar, 2015; Priestley et al., 2015; Leijen et al., 2020; Molla and Nolan, 2020), prompting researchers to examine how relational agency works in university teachers’ professional development especially in regard to enhancing their research capacity.

The majority of the existing literature focused mainly on teachers’ professional communities (e.g., Wang et al., 2017; Chang et al., 2021; Prain et al., 2021). For example, Tao et al. (2020) investigated eight university LOTE (Languages other than English) teachers’ collective agency and relational knowledge development in a multilingual research center. Prain et al. (2021) examined three cases of team teaching and explored the nature of teacher collaboration through strategic use of relational resources. Teachers may develop relationships within and beyond their immediate social and professional milieu (Edwards, 2011), and language teacher agency is perceived as an iterative, dynamic, complex and emergent process (Gao, 2019) as well as spatially and temporally sensitive (Kayi-Aydar et al., 2019). Therefore, more research is needed to expand the understanding of how agency is exercised through the bonding with and support from language teachers’ multi-layered personal relationships at different levels and with differential degrees of impact on their professional development (Tao and Gao, 2017; Vähäsantanen, 2015; Gong et al., 2021). By adopting the personal network perspective to unravel the multi-layered and context-specific relational agency embedded in the constellation of teachers’ personal networks, we may gain a holistic understanding through visual presentations of teachers’ all-round social relationships in a clearer and more direct way.

For more than 40 years, China’s Opening-Up Policy and the recent *Belt and Road* initiative has greatly promoted the Chinese language learning worldwide. Consequently, the rising number of international students has resulted in both long hours of teaching and publication pressure to CSL teachers in the Chinese higher education (Guo et al., 2020). In response, sustained professional development of CSL teachers is crucial at the heart of Chinese language teaching and learning research (Ma et al., 2017; Gong et al., 2020a) and have been extensively discussed both at the institutional and pedagogical levels, including agency research on CSL teachers (Gong et al., 2018a,b, 2020b; Bao et al., 2020; Chang et al., 2021). For example, Gong et al. (2021) investigated the value of agency-oriented approach which fostered Chinese language teachers’ adaptation to online teaching amid the COVID-19 pandemic. Lai et al. (2015) examined how professional identity and social roles shaped agency to enhance professional learning of Chinese language teachers. While the existing studies have yielded useful insights, scant attention has been paid to explore how CSL teachers’ professional development with different learning experiences may develop personal networks and exercise their relational agency (Edwards, 2005; Gong et al., 2020a; Tao et al., 2020). Therefore, this study examines teachers’ all-round social life and hopes to generate insights into the ways in which CSL teachers in a university in China dynamically constructed social relationships and the interactions with their relational agency enactment.

## Understanding Realtional Agency

Relational agency has been conceptualized in a variety of ways. Gergen (2011) views it from the psychological perspective and regards it as a relational phenomenon inherent in actions within relationships. Edwards (2005, 2011) discusses the notion from the socioculturally grounded activity theory to inform the analysis of agency enactment. Further, a growing body of studies investigate teacher agency achieved from the interplay between individual capacity and contextual conditions with agentic choice/action during the spatiotemporal processes (Billett, 2006a; Priestley et al., 2015; Tao and Gao, 2017; Tao et al., 2020). Relations which foster a variety of coordinated activities tend to become the resources to make agency intelligible (Sugarman and Martin, 2011). These relational resources afforded by social networks and relations are highlighted as an essential prerequisite for enhancing teacher agency (Priestley et al., 2015). This helps to expand the conceptual scope by adding the personal network perspective to the present study in order to highlight the role of dynamic social interactions that shape teachers’ social experiences and professional development. We then conceptualize relational agency as involving teachers’ capacity to purposefully interact with others, make choices and take actions to achieve their personal goals. Such a capacity can be developed from capitalizing on different relational resources in the personal networks, which may include projective and intellectual dimensions (Ibarra, 1993; Edwards, 2005; Tao et al., 2020).

Relational agency has a projective dimension. By enacting relational agency, individuals can shape and visualize their future professional goals (Priestley et al., 2015; Hadar and Benish-Weisman, 2019). Human aspirations and the capacity to act may be contingent on the relational context within which people take actions (Annala et al., 2020). Dynamic relationships in the immediate context are likely to initiate and enact individual capacities to articulate shared goals (Pantic, 2015). Moreover, individuals may develop varying degrees of such capacities, and their professional pursuit may accord with their values, choices and aspirations (Hadar and Benish-Weisman, 2019). For example, Chang et al. (2021) demonstrated that Chinese bilingual teachers’ relational agency development tended to promote the goals of bilingual education. The existing literature has so far confirmed the forward-looking nature of relational agency in the way that language teachers may take agentic actions and work toward their valued goals due to the mutually supportive relationships in their personal networks (Kayi-Aydar, 2015; Molla and Nolan, 2020).

Moreover, relational agency highlights the intellectual dimension of interactive learning characterized by critical reflection, increased expertise and capacity to act (Edwards and D’arcy, 2004; Edwards, 2005; Eteläpelto et al., 2013; Feucht et al., 2017). Studies have demonstrated that agentic teachers intentionally interact with others as a resource for learning and are active learners to reflect critically on actions (Billett, 2006b; Pyhältö et al., 2015; Toom et al., 2015; Yang, 2021). Such learning capacity leads to the expanded object such as enriched understanding of the problems and increased expertise which is loaded with intelligence for enhanced action (Edwards, 2005, 2011). Known as scholars of inquiry who engage in research and teaching with critical reflections (Leijen et al., 2020), university language teachers may need to enact relational agency in their research engagement for learning (Priestley et al., 2015; Pyhältö et al., 2015). Such a research process and knowledge production is generally a collective endeavor (Luo and Hyland, 2021), because the nature and extent of the personal relationships that teachers experience is likely to impact on the level of relational agency they enact (Priestley et al., 2015). Therefore, it is necessary to identify and investigate how relational agency enhances university language teachers’ learning capacity and help them become active agents of inquiry.

## Personal Network Perspective

This study adopts personal network perspective as the theoretical foundation to examine the emergence and development of individual teachers’ relational agency. Personal network is defined to place a focal individual (ego) at the center and examine the immediate social relations or a set of people (alters) with whom the individual interacts and connects (Marin and Wellman, 2011; Perry et al., 2018). It does not limit an individual’s relations only to his/her alters but helps to investigate the close connections that the individual identifies so that researchers can explore the in-depth information hidden in the chosen ego-alter ties. By this means, personal network perspective not only ensures the anonymity of any alter that the ego connects but also produce richer, more detailed and nuanced data about each individual’s nascent personal network (Borgatti et al., 2013; Li et al., 2020). For example, Bidart et al. (2018) collected detailed data on young French people in a longitudinal study and build the personal network typology with an in-depth analysis of the vicissitudes and characteristics of their relationships.

Moreover, personal network perspective fits relational agency research and helps to identify the web of social relations that surrounds an individual. According to Crossley (2021), agency is integral and central to the network where interaction, interdependence and relations develops. That is to say, the interplay between agency and the network is supported by the dynamic interpersonal relations and interactions. It is therefore important to investigate an individual’s multi-layered social relations so as to uncover the relational agency enacted from the network. Also, relational agency evolved in the personal networks may influence individual learners and teachers’ academic and professional development significantly. Zappa-Hollman and Duff (2015) found the individual network of practices may provide a research perspective to examine agency and strength of relationships among a group of Mexican students who were actively engaged in the academic English socialization at a Canadian university. Penuel et al. (2012) revealed that personal network helps to identify effective teacher learning and improved practice through various forms of interactions with colleagues inside and outside a writing workshop.

Further, personal network perspective helps to identify the quality, the types and the characteristics of relationships from the network (Levula and Michael, 2016; Rapp et al., 2019; Yang and Markauskaite, 2021). Existing research (Ibarra, 1993) found that personal network has two distinctive types of relationships: instrumental and expressive. Tao et al. (2020) adopted this categorization and found that instrumental relationships rendered teachers intellectual support for knowledge transfer and sharing whilst expressive relationships afforded emotional support. Zappa-Hollman and Duff (2015) elucidated that individual students invested in their individual network in hopes of gaining academic and affective support developing various types of relationships. Therefore, more research is needed to identify the specific types of relationships and the corresponding relational supports in the personal networks and refine our understanding of how these relationships impact on the emergency and development of relational agency.

Collectively, these studies have demonstrated that the existing research mainly focuses on teacher collaboration within the professional learning communities where the strategic use of the relational resources facilitated the enhancement of collective and relational teacher agency (Tao et al., 2020; Chang et al., 2021; Prain et al., 2021). However, few studies have explicitly explored the possible links between relational resources and relational agency extended from professional communities inclusive of teachers’ life world and all-round social experiences (Priestley et al., 2015). Molla and Nolan (2020) argued for the necessity to extend the agency research to a wide set of relationships so as to investigate teacher agency achieved in the context-specific, temporal and relational setting. To this end, our present study tapped into the complexities of the CSL teachers’ professional learning processes during which they constructed their personal networks, capitalized on the relational resources and enacted their relational agency.

Gong et al. (2021) emphasized that CSL teacher agency generally has various manifestations “with multi-levelness and context specificity” (p.87). Therefore, more research is needed to examine different types of social relationships embedded in the constellation of CSL teachers’ professional and social networks, and how these relationships generate resources with which teachers enact their relational agency. To address the above concerns, the present study set out to explore CSL teachers’ relational agency from the personal network perspective. It aims to investigate the diversified ways in which the enactment of relational agency may facilitate CSL teacher professional development and CSL teacher education in different educational contexts. This study thus adopted the personal network perspective which treated the individual CSL teachers as the ego, while the alters were those whom they regarded as resourceful people in their networks. We will address the following research question:

How does relational agency emerge and develop in the CSL teachers’ personal networks?

## Methodology

The study was conducted at the School of Chinese Language and Exchange (SCLE) in a language studies university in China. The school was upgraded from the *Chinese Language Teaching and Research Office* after the incentive of China’s opening-up policy, which resulted in the expansion of student population and the growing demand for CSL teaching and learning. Up to now, the School has offered both undergraduate and postgraduate programs, as the recent *Belt and Road* initiative brought a surging number of students to the school, which has amounted to 500 undergraduate and 280 postgraduate students. The workload per person has reached 14 hours of teaching a week. Moreover, to be promoted to the associate professorship, teachers need to publish at least five international journal articles listed in the Social Science Citation Index (SSCI) or Chinese key journal articles listed in the Chinese Social Science Citation Index (CSSCI) as well as a book. In addition, they have to apply for the research grant at the provincial level. Under such an evaluation scheme, they are confronted with huge pressure of promotion and the tenure track of employment. The present study sought to investigate how these teachers exercise agency and take agentic actions in order to cope with these challenges. Therefore, SCLE was selected as the research site for data collection because it was suitable for us to conduct the investigation into individual CSL teachers’ relational agency.

We adopted purposive sampling method in the data collection and recruited three focal participants on a voluntary basis. Our selection was based on the following criteria: first, these CSL teachers were active and agentic academics. All in their mid-thirties and with doctoral degrees prior to joining this school, they actively engaged with research and teaching by publishing high-quality papers and participating in academic activities. In this sense, they have fully experienced the phenomenon being studied (Creswell and Poth, 2018). Second, they were all pre-tenure novice teachers, and aspired to advance their professional development, and become top scholars in their field of research. Such a shared vision for the furture and imagined professional identity may well demonstrate that they were agentic in taking up their research duties. The following table (see Table 1) presents their basic background information.

**TABLE 1:** Participant profile.

Pseudonym	Age	Weekly Chinese language teaching hours	Research directions	Number of Publications
T1	34	Undergraduates: 8 hours for three courses Postgraduates: 6 hours for two courses	Chinese as second language teaching	5 articles published in journals listed in SSCI
T2	31	Undergraduates: 10 hours for four courses Postgraduates: 4 hours for one course	Chinese phonetic acquisition, Chinese language assessment	3 articles published in journals listed in CSSCI
T3	32	Undergraduates: 8 hours four courses Postgraduates: 6 hours for 2 courses	Chinese language experimental phonology, Chinese phonetics, psycholinguistics	4 articles published in journals listed in SSCI

The study combined the procedures of concentric circles (CC) interviews (Waes and Bossche, 2020) and life history interviews (Creswell and Poth, 2018) for “a qualitative reflection exercise” (Langler et al., 2020, p. 54) in our data collection. Data were collected through conducting three rounds of individual CC interviews with the teachers over three months. Each interview lasted about one and a half hours. We strictly followed the protocol of CC interviews and combined with life history interview questions at times when necessary. Altogether interview hours were 8 hours.

### Concentric Circles Interviews

The purpose was to map out the teachers’ personal networks and delineate the interactions between their evolving relational agency as well as the ways to use these relational resources in the network. While different from other types of social network research that have often been criticized for emphasizing on quantitative features and lacking the examination on the quality of social relations (Bellotti, 2014), CC interviews explore the type, quality and the affective or the instrumental nature of the social relationships. Through visual presentations of the participants’ network, we can therefore identify the relational resources and probe into the dynamic interactions in order to gain a holistic and insider view of the three focal teachers’ relational agency. There are three steps during the CC interview process: (1) boundary setting: the nominalist approach was applied (Langler et al., 2020) to set the boundary specification and focus on the teachers’ immediate relations which contributed to her academic achievements and growth. The first author asked the participants to talk briefly about their educational background and academic experience as a way to collect their academic life history and help them to think about the chosen alters. Then, they were asked to generate the names from the boundary of their personal network and stick the notes of their names on one of the three circles according to the strength of the relations and degree of impact. Finally, they gave the proxy reports about the attributes of the alters on the concentric circles, such as the composition of the relations, the proximity, and types of support. Moreover, they supplied the information regarding the properties of ego-alter ties such as emotional closeness, duration of acquaintance, frequency of contact and degrees of impact (Marin and Wellman, 2011). They further delineated the specific content of their personal relations, vicissitudes and narrative incidents which were perceived and valued as important for them to make agentic choices and take agentic actions in their academic pursuits.

**FIGURE 1:**
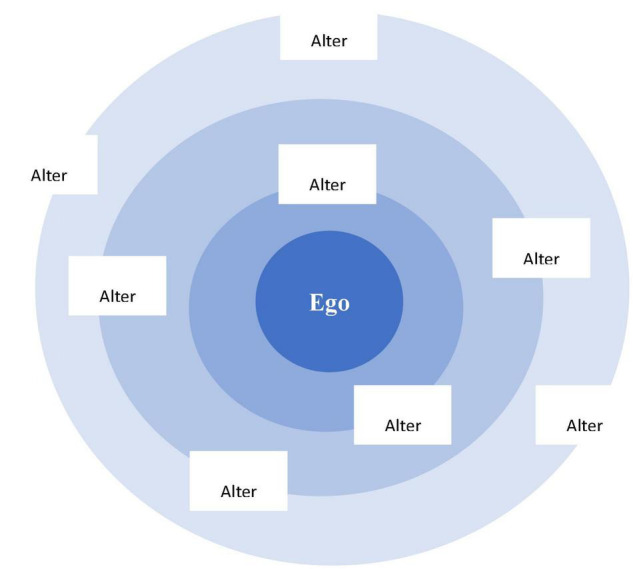
Adapted from concentric circle (CC)map. Source: Van Waes et al. (2015).

In order to follow the procedure, we started with a piece of A3 paper, a pencil and some sticky notes. The piece of paper contained a central dot which represented the participant (the ego) and three layers of concentric circles (Figure 1). The participants were asked to imagine themselves at the center of these circles and then identify and place the stickers on either the inner circle and middle circle. Till the end of the interview, participants were asked to put stickers on the circles. For example, they placed in the innermost circle the names of the alters who had the greatest impact on them. Those whom they did not feel very close but still fairly important were on the middle circle.

### Life-History Interviews

These interviews were self-retrospective and focused on tracking the possible changes in the participants’ personal relations with their chosen alters. The life-course notion of agency may be achieved from the combination of context and time (Eteläpelto et al., 2013; Tao and Gao, 2017). In the form of spatiotemporal space, it is important to explore the choices and actions that they took in the fluid and evolving structure of personal networks. Thus, better understanding was achieved as to how these agentic language teachers constructed their life course by making use of the opportunities and constraints offered from history and immediate social contexts (Emirbayer and Mische, 1998).

### Written Reflections

The participants were also asked to write down their reflections about why and how they developed social relationships with people in their network. Each participant was required to write eight entries of reflections. Altogether there were 24 entries. Through data reduction, 22 entries were used as the data source for triangulation to supplement the missing information about their narratives and kept a record of their various forms of interactions with the people they placed on the concentric circle map (May and Perry, 2014).

The concentric circles interviews combined with life-history interviews were analyzed using thematic analysis to identify agency enacted from various relational resources and the level of impact of these resources on agency enactment (Waes et al., 2016). Then the interview data was converted to three matrices so as to extract and signpost the most salient themes between ego and alters (Miles et al., 2020). Saldana (2016) thematic analytic approaches was used to focus on interrogation, connection, synthesis and abstraction of the data. The first round of concept coding was conducted on the matrices with a focus on the type of support and the development of teachers’ agentic capacity. The broad themes emerged so that the interview data was reorganized into transcript according to emerging themes. The second round of narrative coding analysed the incidents behind the descriptive data. After several rounds of iterative coding, three types of relational resources were identified for agency to develop: value guidance, emotional support and academic support (Table 2). The three researchers read through the codes and themes back and forth several times in order to achieve consensus and to ensure the inter-rater reliability of the data analysis. It is worth noting that while collective agency is often exercised from the colleagial collaborations within the professional communities (Tao et al., 2020), our investigation into participants’ relational agency tapped into the complexities of their all-round social lives beyond the professional milieu.

**TABLE 2:** Relational resources that emerged in the process.

Relational resources	Indicators of enacting relational agency
Value guidance	• Values, beliefs, working attitude, working style • Working attitudes and style as exemplary role model • Praises and encouragements
Emotional support	• Affective empathy • *tucao* (venting and whining) • Life support from family members
Academic support	• Co-publishing • Group study • Supervisors’ academic supervision • Supervising postgraduate students

## Findings

In light of the analysis of the concentric circles and life-history interview data, three types of relationships which the focal participant developed in their personal network were identified, namely, projective relationship, expressive relationship, and instrumental relationship. Moreover, relational resources gradually emerged in the form of value guidance, emotional support and academic support from each type of these relationships. To be more specific, the findings revealed that: (1) the participants were inspired by their previous teachers, supervisors and family members who offered value guidance by sharing their educational philosophy, exemplifying their work ethics, and offering encouragements; (2) they received emotional support from their family members, close friends and colleagues who exerted a positive effect on their emotional well-being; (3) they also received academic support from research fellows who encouraged them to engage in interactive learning through scholarly activities and reflective practices. In this section, the findings will be presented to illustrate how they appropriated these relational resources to their advantage.

### Value Guidance From Projective Relationships

**FIGURE 2:**
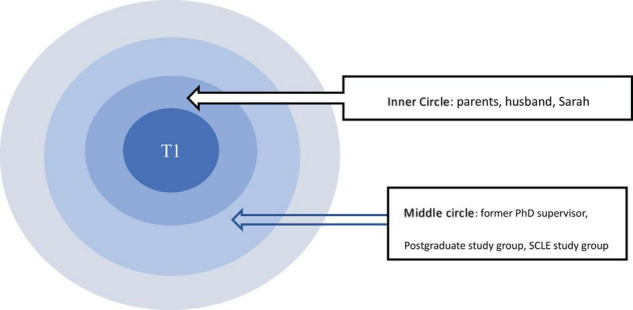
T1’s CC map.

The analysis identified the projective dimension of relational agency as fairly salient in regard to unpacking the dynamic interaction between the resources in the personal networks of the focal participants and the ways they utilized these resources. More specifically, such a projective dimension of relational agency was displayed in the form of value guidance, which can be regarded as a type of relational resource. The participants strategically appropriated and agentively sought these resources in pursuit of their career aspirations. These relational resources were generated from the personal network they constructed from their concentric circles, for example, their parents, previous teachers and doctoral supervisors as well as the faculty dean in the department. These people in their innermost circle greatly influenced their decision making and helped enhance their capacity to act by sharing their value beliefs and educational philosophies, exemplifying their work ethics as role models, and offering constant encouragement. Through such value guidance, the participants developed projective relationships that enabled them to become the type of scholars they aspired to be (Pantic, 2015; Priestley et al., 2015). T1’s concentric circles map in Figure 2 may serve as an illustration. According to the degree of impact, T1 placed husband, undergraduate teacher Sarah, and parents in the inner circle. Former Ph.D. supervisor, SCLE study group and postgraduate study group were put in the middle circle.

First, parents and teacher’s educational philosophy served as the compass to guide the participants’ scholarly pursuit and confirm their decisions of being a university teacher. Figure 2 showed that T1 considered her parents and teacher Sarah as the most inner circle in her personal network, who shared their educational philosophy with her and helped her to choose the scholarly road. She received her parents’ value guidance when she was at life turning points, such as choosing the university major and continuing her postgraduate study. She benefited from their educational philosophy and enjoyed the freedom of life decisions:

[1] My parents are liberal and supportive. They believe interest gives people everlasting learning momentum. Most parents persuaded their children to major in economics or finance which seemed have a good job prospect. But my parents cared most where my interest located. I am fond of Chinese, history and geography. With their support I chose Chinese language education as my major (CC interview).

Another influential person to T1 is Teacher Sarah, who taught a year-four undergraduate course *Critical Reading* and offered advice to T1 on her decision to continue her postgraduate study. T1 reflected in the following excerpt that she had no idea of critical reading and thinking before taking Sarah’s course as she was the first to guide students to challenge and critique the published articles:

[2] Sarah’s own research was groundbreaking. She was a teacher with charisma, talking with sense and bringing students new perspectives and enlightening ideas. Upon graduation, I found I had many potential research topics, so I decided to carry on postgraduate study. Up to now, she still influences my teaching to be pin-pointing and evidence-based (life history interview).

Similarly, T2 shared with us her appreciation of her father’s firm belief in the values of academic work and much encouraged by her father’s perception of women’s role in the academia:

[3] My father works in university as a scholar himself. He gives credit to this profession. That’s why he fully supported me to be university teacher. He has high hopes on my academic achievements. Other parents may think when their daughters reach thirties, they should give birth first. My father never thinks so. He asked me not to let my academic work delayed by bearing a child (life-history interview).

Second, the participants’ previous Ph.D. supervisors set an exemplary role model for them, because they shared their work ethics that underscored the importance of being serious toward teaching and research. For example, both T3 and T2 both highlighted the word “seriousness” during the interviews to describe their supervisors’ working style.

[4] T3: I was strongly influenced by my supervisor’s serious working attitude. He was the elite scholar in our field, because he was extremely hardworking and was the most meticulous person. When he read my draft, he even corrected my punctuation and article use (CC interview).

[5] T2: My supervisor was a serious person at work. He discussed with me the problems in my research project and taught me how to revise. We kept close and intensive interactions. I had to keep up with his serious working style and dared not be lazy (CC interview).

The above excerpts showed that the participants appreciated their work ethics and followed their value guidance to fulfill the tasks. The projective relationship thus shaped their work ethics and motivated them to become serious and responsible scholars.

Moreover, the positive verbal confirmations from the participants’ supervisors or the faculty dean also offered guiding values. For example, T3 mentioned being grateful for the dean’s encouragement toward early-career teachers’ enthusiasm in research as the CSL teachers in the department had fairly heavy teaching loads and usually did not have much time for research. She felt encouraged to engage actively in research. Therefore, the encouragement from supervisors and the dean generated their working momentum, which in tandem evoked their agentic actions for research progress.

The above analysis showed that as a type of relational resource, value guidance conduces to the enactment of relational agency, which ignited participants’ aspirations and enabled them to visualize themselves as becoming their aspired scholars (Hadar and Benish-Weisman, 2019). In this regard, the findings confirmed the importance of the projective dimension of relational agency (Pantic, 2015; Priestley et al., 2015; Annala et al., 2020) as the participants became agentive to make decisions and take actions when being directed and inspired by the people who shared their educational philosophy, exemplified their work ethics, and offered encouragements.

### Emotional Support From Expressive Relationships

**FIGURE 3:**
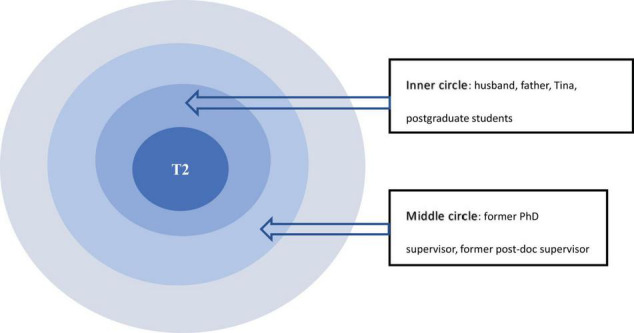
T2’s CC map.

The data revealed that emotional support was flowing through expressive relationships among the participants’ personal networks (Ibarra, 1993; Waes and Bossche, 2020; Yang and Markauskaite, 2021). Emotional support was manifested in the network of kinship, close friends and colleagues. As an evident and important type of relational resource, emotional support was offered through affective empathy, *tucao* (Tao et al., 2020) and life support from family members which had a positive effect on participant emotional well-being. These forms of support may empower the participants to enact their relational agency in expressive relationships. T2’s CC map in Figure 3 showed that husband, father, and close friend Tina were in the inner circle with the highest degree of impact on her, while former PhD supervisor, former post-doc supervisor at the middle circle were also important relational ties to her.

First, the data showed that empathy was usually rendered by those who fully understand participants’ hardships. Figure 3 demonstrated that empathy from people in the innermost circle of her personal network impact her to the greatest extent. These people included her husband and one of her closest friends. She reflected in the interview that her husband’s support was empathetic and touching. He was also a university teacher and understood the hard times she had been through. She considered herself as extremely lucky to have such a husband as being able to share all the ups and downs on the journey:

[6] When I got rejections from journals, my husband’s comfort was not that important. I have already been used to these failures. But when I successfully published articles and won the research funding, my husband was truly happy for me. That was the biggest comfort to me, because he knew how much this success meant to me. He understood the deep value of my hard work (CC interview).

The expressive relationship as shown in the above excerpt was built on the basis of their shared understanding of the situations. The empathy from her husband helped T2 to confirm the value of academic work and further enact her agency to strive for the challenging yet rewarding academic profession.

Furthermore, Figure 3 also showed that T2’s high school classmate Tina was another relational resource of her emotional support. They came to the same city for undergraduate studies, which provided them opportunities to be close to each other. But what bonded them was their goal-oriented and enterprising spirits. T2 said in the following excerpt that though they were in different professions, they both had their career ambitions:

[7] We set a goal for ourselves that both of us had to achieve something in our professional areas. When we met, we often talked about our work and what plan we would make for the next step. I cherish to have a friend like her (CC interview).

The empathy between T2 and Tina was mutually engaging. They shared the same working style as being hardworking and enterprising. Their friendship continued and emotional reliance grew stronger on the common ground. T2 confessed if she had not had Tina’s friendship, she would have not been so agentic. As such, her relational agency was well sustained.

Second, when the participants confronted failures, challenges and pressure, complaining or tucao was only a temporary reaction because they seemed to be able to dilute their negative emotions and took positive actions in response to the setback quickly. For example, the three participants and the other two male young teachers from SCLE formed a study group. In face of the challenging university promotion policy, they were all anxious to get promoted to the associated professorship, but none of them succeeded. Though they complained about the policy, they soon found complaints were useless and got down discussing the ways to meet these tough requirements. Relational agency was thus enacted by taking concrete actions.

Third, family members in the innermost circle of network reduced the participants’ emotional burden to a great extent and made sure they were able to keep engaged in their research practices by helping with the household chores and problems. For example, T1 shared that her husband took good care of the family so that she was able to concentrate on the revisions of her paper:

[8] I told him I received a feedback from the journal and had to finish major revisions within a month. He supported me without any complaint, took care of our 8-month-old daughter and did all the housework. This was the most invaluable support to me as a woman researcher. My nerves were greatly pacified. I then completed the task in due course and got the paper published (life history interview).

Such support from the husband also made the participants feel respected. T1 was sent by the faculty to teach Chinese in the Overseas Confucius College in the United States. Her husband was again supportive for her decision to leave the family for 1 year, as he knew it would benefit her professional development. T1 confessed such respect was precious to her, and served as the bedrock which helped solve real problems and offered encouragement.

The findings revealed that expressive relationships may generate agency for teachers to release from anxiety and move toward their goals with agentic actions. It is worth noting that *tucao* was identified within a teacher learning community as the instant emotional comfort. The emotional support in the present study extended from the whining and venting form of *tucao*, which played a significant role in enacting participants’ relational agency. Participants utilized relational resources through *tucao* of this type for affective empathy and practical solutions in response to different challenges.

### Academic Support From Instrumental Relationships

**FIGURE 4:**
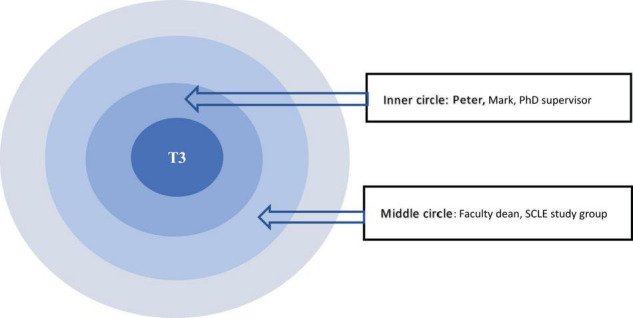
T3’s CC map.

The data revealed that the intellectual dimension of relational agency was specifically enhanced by different types of academic support as a dynamic form of relational resource in participants’ networks to shape instrumental relationships. Academic support encouraged interactive learning (Edwards, 2005) when the participants were engaged in research collaboration, group study and academic supervision. These scholarly activities (see Figure 4) constructed a mutually constitutive personal network by means of sharing and building their distributed intelligence and expertise (Edwards and D’arcy, 2004; Yang, 2021).

First, research collaboration connected the participants with coauthors by making joint efforts to meet deadlines and fulfill the tasks. They were able to enhance their relational agency by combining their distributed intelligence. For example, T3’s collaboration with her co-authors exemplified how she received academic support. Peter was her postgraduate classmate who was conducting research in psycholinguistics while T3 specialized in the Chinese phonetics. They combined their expertise and used the psycholinguistic approach to investigate the Chinese tones. They managed to publish two journal papers using the interdisciplinary perspective. Likewise, T3 worked with her boyfriend Mark in the same way by expanding their research scope across interdisciplinary fields. Mark worked in the theoretical construction of linguistics with no experience in conducting empirical research, so they combined their research expertise and published an article. Thanks to their swift actions and fast working pace, their research collaboration went very well, as T3 shared in the interview:

[9] Peter and I are both quick in action and collaborate for clear shares of tasks. He wrote the literature review, and I finished the experiment and wrote up the research findings. We then worked together to write the discussion for a few days. Then the first draft was done. Mark is remarkably swift in action. I can’t write when I am occupied with different tasks and need a week free of any burden to write. Mark just sits down and write right away. Both of us have our own working pace (CC interview).

Figure 4 showed that Peter and Mark were in the innermost concentric circle of T3’s social network and relational agency was enacted through the goal-oriented collaborations, well-distributed research expertise and compatible working pace. This mutually constitutive relations helped them to make agentic choice and actions (Billett, 2006a; Toom et al., 2015).

Group study enhanced interactive learning and collaborative inquiry where expertise was effectively distributed and shared within the groups. T2 said she benefited from her postgraduate group study:

[10] In the group, every one has to share their own research expertise. I used to present how to use SSP for variance analysis. Some group mates were good at statistics of non-parametric tests and taught us this analytical skill. I stayed in the group after graduation and my research knowledge was updated timely. Recently some group mates have presented the technology of brain electromagnetism; Some shared ERP statistical analysis. We often discuss heatedly during the meetings. Last month I shared my experience of how to write English articles (life history interview).

These interactive learning activities helped to expand the knowledge so that relational agency was enhanced by active participation of sharing and acquiring new skills and technology. In addition, more than knowledge acquisition, group study also shaped inquiry mind and scholarly thinking (Leijen et al., 2020). For example, T1 found that the group study in postgraduate years cultivated her thinking ability:

[11] Three years of postgraduate study influenced me a lot. Every Tuesday we had group study. We had to report what articles we read and discover the deep meaning embedded in the article. I was trained to articulate my thoughts, ask questions, inquire into problems and deepen my understanding. My logic was intensively trained during that time (life history interview).

The study groups provided a favorable environment for knowledge building and intellectual progress. These instrumental relationships involved participants in profound interactions to share expertise and shape an inquiry mind. Thus, relational agency was enhanced in a way that the participants become active learners and researchers.

In addition, the data revealed that the participants kept frequent contact with their previous doctoral supervisors and developed instrumental relationships with them because the academic support from their supervisors helped cultivate their research capacity in the long run and they benefited greatly from critically reflect on their learning. For example, T1 and T3 in the life history interviews talked about their supervisors’ strategic guidance. They did not tell the students what to research but let them search on their own. Their patient guidance and strict tutorials finally helped the students identify their research topics. T1 then followed the same style and supervised her own postgraduate students to explore research questions step by step. She argued if the supervisor told the students directly what topic to do and what measures to take, it was quick to complete the task while the students may be left in the mist without fully understanding the subject:

[12] When I guided them strategically, they would form the habit of inquiry and delve into the deep meaning. They might go the wrong way but they would learn from their mistakes and be clearer about what they were targeting (life history interview).

Further, both supervisors and students can improve their academic ability in tandem in the instrumental relationships. T2 regarded supervising postgraduate students as an experience of growing to be a real scholar:

[13] When students decided to do a project, I had to take time to learn and assess the validity of the project. This is challenging but motivating. Also, when students had a different research finding, I needed to find out why they gained different results. This inquiry process was motivating, too. Sometimes their writing was unclear. I had to research what caused them to understand in a different way. After this exploration, I would gain much new insights (CC interview).

The above data showed that the process of being supervised and supervising others engaged the participants in reflective practices (Leijen et al., 2020) and improved their own research capacity. Relational agency was enhanced through research engagement and accumulation of expertise with concerted efforts. Instrumental relationships helped to connect intellectual beings in a mutually constitutive and supportive way. Their reciprocal and collective endeavor became the relational resources not only to shape and strengthen instrumental relationships but also to make agency intelligible and enhanced (Sugarman and Martin, 2011).

## Discussion

The present study investigated how three focal CSL teachers exercised their agentic choice/action in their personal networks as being cultivated and empowered to become reflexive and reflective agents for their sustainable professional development. Three types of relational resources were identified through our analysis of their concentric circle networks. These resources were manifested in the form of value guidance, emotional support and academic support, which emerged from projective, expressive and instrumental relationships respectively. These findings confirmed with the previous studies that such relationships highly facilitated dynamic interpersonal interactions (Gergen, 2011; Priestley et al., 2015; Pyhältö et al., 2015; Hadar and Benish-Weisman et al., 2019). Individual language teachers thus agentively appropriated these resources in different ways to enhance their agentic choice/action capacity through these interactions (Billett, 2006a; Toom et al., 2015). The study contributes to the teacher agency literature in three major ways: It incorporates the method of using concentric circles interviews as one of the predominantly qualitative social network analyses into relational agency research and offers a more articulate picture of the complex mechanisms behind the agency development. The second unique feature is that the research scope has been expanded to examine the CSL teachers’ all-round social life and experiences beyond the traditionally bounded professional community, and therefore achieved a more nuanced and refined understanding of the emergency and development of relational agency mediated by contextual conditions. More importantly, a concentric circles model of individual teachers’ relational agency is proposed in an effort to establish an explicit conceptual link between the resources and types of support embedded in the social relationships with relational agency. Future empirical research may consider building on such a model for investigations into language teachers in different societal contexts.

While resonating with Edwards (2015) advocacy for exploring teacher agency from relational and collective perspectives, the study contributes to the literature by expanding the conceptual understanding of relational agency, and argues that relational agency tends to go beyond the psychological interpretation of personal dispositions and the physical boundaries of language teachers’ professional communities (Edwards and D’arcy, 2004; Chang et al., 2021). By taking into a fuller consideration of the role that all the important people and resources in their personal social network may play, we delved into depth the different ways that individual teachers capitalized on a diversity of relational resources to enhance their agentic choice and actions as well as the capacity to interact with others (Priestley et al., 2015). The findings suggest that personal network may play a critical role in the development of relational agency to sustain language teachers’ professional growth.

First, the study identifies an emerging new type of relational resource in the projective relationship that the participants developed in their social network, in addition to instrumental and expressive relationships (Ibarra, 1993). The findings lend empirical support to the projective dimension in the ecological model of teacher agency (Priestley et al., 2015) and answers the call of Molla and Nolan (2020) to extend the agency research to a wide set of relationships across the boundaries among teachers’ professional and personal networks. Our analysis has demonstrated that the projective relationship plays a fairly salient role because the enactment and enhancement of relational agency may be mediated by the value guidance from this type of relationship with parents, previous supervisors and the faculty dean in the innermost circle of the participants’ network. By sharing their own values and aspirations (Annala et al., 2020), these people provided guidance oriented toward the future by imparting their educational philosophy, exemplifying their work ethics, and offering encouragements. The study suggests that the personal network may help visualize a diversity of projective relationships and thus supports Hadar and Benish-Weisman (2019) that values and aspirations relate to agentic capacity and can be applied to enhance teachers’ agentic behavior and actions and consequently reshape their identity commitment as to become aspired scholars (Priestley et al., 2015; Tao and Gao, 2017).

Second, while echoing Zappa-Hollman and Duff (2015) identification of affective support in the individual network, our study further unveiled “the inherently social, affective, and human nature of the networks” (p. 360) through the combined procedures of concentric interviews and life history interviews. We argue for an expansive form of emotional support provided timely by the participants’ family members, close friends and colleagues. These people in the expressive relationships shared their understanding of and respect for language teachers’ work situation and demonstrated considerable empathy toward them. For example, T2’s husband understood the deep values of her academic work; her best friend Tina strongly aligned with her working spirits as being goal-oriented and enterprising. The empathetic nature of the emotional support emerged from their concentric circles greatly encouraged them and well sustained their relational agency. The findings also complement Tao et al. (2020) taxonomy of *tucao* in the way that teachers may go beyond merely venting and whining about things by resorting to the relational resources and adopting practical strategies to cope with challenging situations. Consequently, they were able to release pressure and felt emotionally supported at the same time. Additionally, our study indicates that family members’ timely and practical solutions of some urgent problems in life provides these teachers with an alternative form of emotional support so that they can stay focused on research without being distracted. Such action support has reduced the female teachers’ dual duties of having to usually juggle with work and effectively enhanced their emotional well-being for robust academic growth.

Moreover, the findings have highlighted the importance of collaborative inquiry between the language teachers and the people in their personal network, which may considerably enhance their agency to engage in research practices by sharing distributed expertise, building knowledge together and developing the ability to ask critical questions (Edwards and D’arcy, 2004; Toom et al., 2015). Through such collaborative inquiry, they received academic support from the instrumental relationships that they developed in their concentric circles network. For example, T3 was highly motivated by research collaborations with Peter and Mark and managed to publish papers in journals. Nevertheless, such interactive learning does not remain at the level of knowledge building and expertise exchange but are extended to engage teachers in cultivating their inquiry ability. Both T1 and T2 reported their critical thinking and research capacity were improved because of their active participation in group studies and academic supervisions. The study highlights the importance of critical reflective practices on learning as being emphasized by Leijen et al. (2020) who called for effort to strengthen the conditions for achieving teacher agency. Furthermore, in accordance with Luo and Hyland (2021) tenet to assist researchers to identify and use networked resources, the findings point to the need for language teachers to consider developing a social network from which they seek potential research collaborators and construct instrumental relationships that may provide them with immediate academic affordances. Thus, we may reasonably speculate that with these affordances and resources, teachers can be agentively engaged in the process of collaborative inquiry that may go beyond merely knowledge building but more importantly, cultivate them to be active agents of inquiry.

**FIGURE 5:**
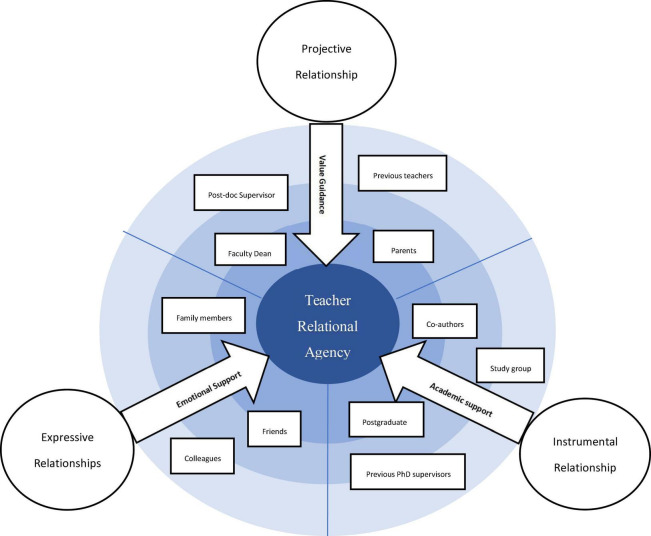
A concentric circle model of relational agency.

In light of the above findings, a concentric circle model of individual teachers’ relational agency is proposed in Figure 5 that reflects the dynamic interaction between language teachers’ relational agency and their personal network. The three concentric circles in different shades denote the strength of the relations and degrees of impact, which are imbued with the dynamism of individual teachers’ social participation and interactions. The inner circle signals the most intensive degree of interaction and the strongest impact, whilst the middle circle and the outer circle are of relatively lower level of interaction and impact.

As displayed in the model, three types of relational resources are manifested in the form of value guidance, emotional support and academic support and may emerge from the projective, expressive and instrumental relationships respectively. Value guidance is usually provided by people with a high social and academic status as well as abundant social experiences so that they can impart their educational philosophy, exemplify their work ethics, and offer encouragements to enhance language teachers’ agentic capacity (Annala et al., 2020). Emotional support may be provided by their family members or people who can understand the value of their work, respect their work situation, demonstrate considerable empathy and offer action support. Relational agency achieved from this type of support not only sustains teachers’ emotional well-being but also enhances their agentic actions to achieve goals. People who provide academic support offer research assistance for collaborative inquiry may help enhance their agentic capacity to engage with other academics for knowledge sharing and research collaborations. No matter what relational resources individual teachers appropriate and utilize, the model can effectively visualize and conceptualize how relational agency emerges and develops through multi-layered constructions of relationships. Future research may consider using this model to continue the inquiry into language teacher relational agency situated in individual teachers’ personal networks.

## Conclusion

This study explored the ways to empower CSL teachers to achieve their academic goals and sustainable professional development through the theoretical lens of relational agency enactment in the personal networks. More specifically, it examined the multi-layered relational resources embedded in the concentric circles network that constitutes their professional communities and life world. Moreover, the type, extent and nature of these relationships in terms of the degree of relational impact was investigated. In light of the findings, the projective, affective and instrumental dimensions of the social relationships were identified, from which relational agency gradually emerged from the language teachers’ engagement in the interpersonal interactions.

The study has thus demonstrated the conceptual and analytical strength of personal network in teacher relational agency research that contributes to our understanding of language teachers’ relational agency. It provides practical implications for language practitioners and educators to understand how to enact and enhance relational agency so as to sustain their professional development. The findings also confirm that such efforts are fairly important because fostering appropriate relationships in teachers’ professional life has become a prerequisite for teacher agency to emerge and develop. Language teachers are encouraged to interact with people who may render value guidance, emotional and academic support so that these relational resources not only shape conducive relationships but also enhance their agentic capacity to make decisions, meet challenges and agentively fulfill their academic aspirations.

We thus call for more efforts to purposefully enhance relational agency of university language teachers by creating opportunities and spaces for quality interpersonal interactions so as to empower them and build their career as aspired academics. More than that, separate attention and efforts should also be paid to university CSL teachers in order to generate theoretical and practical implications for relational agency research. By so doing, we hope CSL teachers’ professional competence can be enhanced and their identity commitment as scholars in response to various educational changes reshaped. In this sense, the research may contribute to the limited and even peripheral CSL teacher agency research by deepening our understanding of Chinese language teachers in the local educational context.

This study is a retrospective small-scale exploration based on the research of three CSL teachers’ personal networks. As the findings may not be generalized to a wider scope of academic lives, future studies are to be conducted on CSL teachers as well as other different types of language teachers in a variety of pedagogical contexts including teacher education and development programs in order to gain a holistic understanding of the development of language teacher relational agency. It is nevertheless hoped that the present study will encourage university CSL teachers and other language teachers become resourceful academics with agentic capacity to take initiative and participate in social interactions so as to enhance the dynamic interplay between relational agency and the immediate social context.

## Data Availability Statement

The original contributions presented in the study are included in the article/supplementary material, further inquiries can be directed to the corresponding author.

## Ethics Statement

The studies involving human participants were reviewed and approved by Shanghai International Studies University. The patients/participants provided their written informed consent to participate in this study.

## Author Contributions

WY conceived and designed the study, collect and analyzed the data, and wrote the manuscript. CL conceived, designed the study and wrote the manuscript. XG conceived the study and offered suggestions for revisions. All authors contributed to the article and approved the submitted version.

## Conflict of Interest

The authors declare that the research was conducted in the absence of any commercial or financial relationships that could be construed as a potential conflict of interest.

## Publisher’s Note

All claims expressed in this article are solely those of the authors and do not necessarily represent those of their affiliated organizations, or those of the publisher, the editors and the reviewers. Any product that may be evaluated in this article, or claim that may be made by its manufacturer, is not guaranteed or endorsed by the publisher.
